# Levocarnitine mitigates varicocele-induced spermatogenic impairment by restoring mitochondrial integrity and redox homeostasis in rats

**DOI:** 10.3389/fcell.2026.1783995

**Published:** 2026-07-14

**Authors:** Gang Ma, Chuang Huang, Bin Zhang, Kai Zhang, Fudong Li, Liuting Xu, Weijun Zhang, Dehui Chang

**Affiliations:** 1 Department of Urology, The 940th Hospital of the Joint Logistics Support Force, Lanzhou, Gansu, China; 2 Nanyang Second People's Hospital, Nanyang, Henan, China

**Keywords:** catalase, FoxO3a, L-carnitine, mitochondrial ultrastructure, oxidative stress, SOD2, spermatogenesis, varicocele

## Abstract

**Background:**

To study the protective mechanism of L-carnitine on the reproductive function of varicocele Wistar rats through FoxO pathway.

**Methods:**

Forty 8-week-old male Wistar rats were randomly divided into four groups (n = 10): control group (A), sham-operated group (B), model group (C) and L-carnitine group (D). Varicocele was induced in Groups C and D via partial ligation of the left renal vein. Post-modeling, Group D received daily intraperitoneal L-carnitine (100 mg/kg), while Groups B and C were administered equivalent volumes of saline. After 28-day, we evaluated the modeling efficacy and measured the following parameters: body weight; epididymal and testicular indices (organ weight/body weight × 100%); semen quality (count, motility, morphology); testicular histomorphology and ultrastructure; DNA damage; and protein expression levels of FoxO3a, CAT, and SOD2.

**Results:**

Relative to controls, model group rats exhibited: (1) significantly reduced testicular index (P < 0.01), (2) impaired spermatogenesis (decreased sperm density, viability, and progressive motility [PR%]; P < 0.01), (3) disrupted seminiferous tubule architecture, (4) lower Johnsen scores (P < 0.001), (5) severe mitochondrial ultrastructural damage, and (6) elevated oxidative stress (increased MDA, 8-OHdG, and ROS with concomitant SOD reduction; all P < 0.05). L-carnitine treatment significantly alleviated varicocele-induced impairments and partially reversed oxidative stress and DNA damage–related alterations (P < 0.05), indicating its potential testicular protective effect through oxidative stress mitigation.

**Conclusion:**

Our results indicate that L-carnitine partially restores male reproductive function in varicocele models by improving sperm quantity and quality, likely through its antioxidant properties and potential involvement of the FoxO3a signaling pathway.

## 1 Introduction

Varicocele refers to the microangiopathy of spermatic vein plexus dilatation and blood stasis caused by spermatic vein valve insufficiency or poor venous return. It is the most common cause of male infertility and male semen quality decline (Punab et al., 2017; Leslie et al., 2022). The main pathological mechanism may be oxidative stress. Under normal circumstances, the veins around the testis discharge heat and waste from the testis, while the blood stasis in the spermatic vein can lead to overheating and hypoxia of the testis, resulting in excessive reactive oxygen species (ROS) (Wang et al., 2022; Lv et al., 2022; Chakraborty and Roychoudhury, 2022) while producing mitochondrial energy. A small amount of ROS produced in the mitochondria can be removed by the antioxidant defense system composed of superoxide dismutase-2 (SOD2) and catalase (CAT) in the mitochondria. However, when the body continuously produces excessive ROS for a long time, which exceeds the total antioxidant capacity of the antioxidant defense system, it will adversely affect the occurrence and maturation of sperm, causing male infertility (Moretti et al., 2021; Santana et al., 2020; Erfani Majd et al., 2019).

At present, the clinical treatment of varicocele is mainly various surgical treatments, among which microsurgery is the most effective treatment (Ding et al., 2012; Cayan et al., 2009; Wang and Ji, 2020). However, some studies have found that it is not effective for patients with high grade varicocele (Asafu-Adjei et al., 2020), and there is excessive medical risk in preventive surgery for adolescent patients (Locke et al., 2017). Varicocele mainly leads to excessive ROS in the testis, affecting sperm motility, acrosome reaction and chromatin maturation, resulting in increased DNA fragmentation, resulting in decreased sperm quality and male infertility (Sidorkiewicz et al., 2017). Therefore, the use of antioxidants is a feasible method, and vitamin C, levocarnitine (L-carnitine) and other antioxidants are currently used in clinical treatment. However, due to the lack of high-quality basic and clinical drug research, the efficacy of various antioxidants, including L-carnitine, is still controversial. There is no clear guideline-recommended antioxidant treatment for male sperm quality decline caused by varicocele. (de Ligny et al., 2022; Showell et al., 2014; Steiner et al., 2020). Therefore, it is of great practical significance to carry out safe and effective antioxidant drug prevention and treatment of male semen quality decline caused by varicocele.

In the male reproductive system, L-carnitine is enriched in the epididymis and plays a crucial role in sperm metabolism and maturation. They are associated with sperm motility and have antioxidant properties (Agarwal and Said, 2004; Niederberger, 2005). Researchers have found that there is a statistically significant positive correlation between semen L-carnitine level and sperm concentration, motility and normal morphology (Mostafa et al., 2022). L-carnitine in seminal plasma can also inhibit macrophages to protect sperm membrane and chromatin from free oxygen free radicals by mimicking the role of glucocorticoids (Ma and Sun, 2022). However, most studies lack placebo control and double-blind design, so it is difficult to draw a clear conclusion. Therefore, using L-carnitine as an intervention drug and studying its mechanism of action is of great significance to the oxidative damage of the male reproductive system caused by varicocele.

In this study, a male Wistar rat model of varicocele was prepared by partial ligation of the renal vein. The L-carnitine group was compared with the blank group and the model group to study whether L-carnitine has a scavenging effect on the excessive production of ROS in mitochondria caused by varicocele and its possible mechanism. To explore and clarify the efficacy and mechanism of L-carnitine on reproductive injury caused by varicocele. Here, we evaluate whether L-carnitine alleviates varicocele-induced oxidative stress and mitochondrial injury in the testis and explore the potential involvement of the FoxO3a-associated antioxidant response.

## 2 Materials and methods

### 2.1 Animal model establishment, grouping and management

Forty healthy male Wistar rats, 8 weeks old, weighing 200 ± 20 g, were provided by the 940th Hospital of the Joint Logistics Support Force (License No.: SYXK (Military) 2017-0047). This study has been approved by the Ethics Committee of the 940th Hospital of the Joint Logistics Support Force of the Chinese People’s Liberation Army (Approval No.: 2021KYLL143). The experimental animals were fed in the animal experimental department of the 940th Hospital of the Joint Logistics Support Force. The feeding temperature was 22 °C–24 °C, the humidity was 35%–50%, and the light-dark cycle was 12 h. The automatic feeding system was supplemented with feed. Forty rats were randomly divided into control group (group A), sham operation group (group B), model group (group C) and L-carnitine group (group D), with 10 rats in each group. Group A did not perform any operation; in group B, only the fascia around the left renal vein was separated, and the left renal vein was exposed without ligation. Varicocele was induced in Groups C and D by partial ligation of the left renal vein, as previously described (Najari et al., 2014). Successful modeling was confirmed by visual observation of dilated and tortuous left spermatic veins with a diameter >1 mm and no obvious atrophy of the left kidney (Cao et al., 2013). The 28-day intervention period was selected based on the spermatogenic cycle of rats (approximately 48–53 days), allowing coverage of at least one partial spermatogenic cycle. This duration is consistent with previous studies investigating varicocele-induced testicular dysfunction and antioxidant interventions in rodent models.

### 2.2 Testicular organ index

On the 28-day, the rats were fasted for 24 h after the last administration. The rats in each group were anesthetized by intraperitoneal injection of 10% chloral hydrate (3 mL/kg). After weighing, the size of bilateral kidneys was observed after opening the abdominal cavity, and the diameter of left spermatic vein was measured by vernier caliper. After judging the success of modeling, the left testis and epididymis were completely removed from the scrotum, and the surrounding adipose tissue was removed. After weighing and recording the data, they were fully cut into pieces and placed in a pre-prepared constant temperature normal saline (37 °C) for later use.

Testicular organ index = (testicular weight (g)) / (rat body weight (g)) × 100%.

### 2.3 Observation of epididymal sperm activity rate, deformity rate and sperm count

The fully cut epididymal tissue was placed in 4 mL of 37 °C constant temperature normal saline, 37 °C water bath for 25 min. After the epididymal sperm was fully free, 1 mL was drawn for standby. The remaining free sperm was detected by Weili digital color sperm quality detection system (Beijing Weili New Century Technology Company, WLJY-9000). Sperm activity rate and deformity rate; the 1 mL sperm suspension was diluted 5 times with normal saline again. After fully mixing, 1 mL was dripped into the red blood cell counting plate and counted under the microscope (Leica, Leica DM2500, Germany).

### 2.4 HE staining was used to observe the morphology of left testicular tissue in rats and johnsen score was performed

Testicular tissue fixed in 10% formaldehyde solution was cut into about 5 mm thick tissue slices, dehydrated with 75%, 85%, 90%, 95% ethanol and anhydrous ethanol in turn, soaked in xylene, embedded in paraffin, cut into 4–6 μm thick slices, flattened and placed on glass slides. After fixation, dewaxing, dehydration, washing, hematoxylin staining, 85%, 95%, 100% alcohol gradient dehydration after eosin counterstaining, and then transparent with xylene again. Finally, the slices were sealed with neutral gum, observed under an optical microscope, and scored according to Johnsen‘s scoring standard. Data analysis was performed on the Johnsen score. All histological scoring (Johnsen’s score) was performed by two independent investigators in a blinded manner.

### 2.5 Preparation of testicular homogenate and detection of SOD, MDA, ROS, 8-OHdG

The testicular tissue stored at − 80 °C was weighed and placed in a cryopreservation tube, and steel balls were added. The frozen high-throughput tissue grinder was uniformly ground. After grinding, normal saline was added according to the ratio of testicular weight (g): normal saline volume (mL) = 1 : 9, and the supernatant was 10% testicular homogenate after centrifugation. The BCA protein concentration kit (Beijing Suolaibao, PC0020) was used to determine the protein concentration of testicular homogenate supernatant according to the instructions. The content of SOD in testicular tissue was detected and calculated according to the instructions of SOD determination kit (Nanjing Jiancheng Bioengineering Institute, A001-3). The content of MDA in testicular tissue was detected and calculated according to the instructions of MDA detection kit (Nanjing Jiancheng Bioengineering Institute, A003-1-2). The content of ROS in testicular tissue was detected and calculated according to the instructions of ROS assay kit (Nanjing Jiancheng Bioengineering Institute, E004-1-1). The content of 8-OHdG in testicular tissue was detected and calculated according to the instructions of 8-OHdG DNA damage quantitative kit (Beijing EpigenTek Reagent Co., Ltd., P-6003-48).

### 2.6 The expression of FoxO3a, CAT and SOD2 protein in testicular tissue of rats in each group was detected by western blot

Testicular tissue was homogenized in RIPA lysis buffer containing protease and phosphatase inhibitors. Total protein concentration was determined using a BCA protein assay kit. Equal amounts of protein (30–50 μg) were separated by SDS-PAGE and transferred to PVDF membranes. After blocking, membranes were incubated overnight at 4 °C with primary antibodies: anti-FoxO3a (Abcam, ab12162, 1:1000), anti-CAT (Cell Signaling Technology, #12980, 1:1000), anti-SOD2 (Abcam, ab13533, 1:2000), and anti-β-actin (as loading control, 1:5000). After washing, membranes were incubated with HRP-conjugated secondary antibodies (1:5000) for 1 h at room temperature. Protein bands were visualized using ECL reagent and quantified by ImageJ software. Relative protein expression was normalized to β-actin.

### 2.7 Observation of spermatogenic cells and supporting cell organelles in testicular tissue by transmission electron microscopy

The testicular tissue fixed in the electron microscope fixative was taken out, rinsed with phosphate buffer, fixed in osmic acid, rinsed with phosphate buffer again, and dehydrated with 30%, 50%, 70%, 80%, 95%, 100%, 100% ethanol and 100% acetone in turn. The samples were inserted into the embedding plate and embedded in the oven at 37 °C overnight. The sections were stained with 2% uranyl acetate saturated alcohol in the dark. After washing with 70% alcohol and ultrapure water, 2.6% lead citrate solution was counterstained. After washing with 70% alcohol and ultrapure water, the samples were dried overnight at room temperature. Ultrastructural analysis was conducted by two independent investigators blinded to the experimental groups.

### 2.8 Single cell comet electrophoresis experiment

Testicular single-cell suspensions were prepared and mixed with low-melting-point agarose. After lysis, DNA unwinding, and electrophoresis under alkaline conditions, slides were neutralized and stained with propidium iodide (PI). Images were captured under a fluorescence microscope. Tail length (TL, the distance from the center of the head to the end of the tail) and Olive tail moment (OTM, the product of the tail length and the fraction of total DNA in the tail) were used as indicators of DNA damage. One hundred cells were randomly selected and analyzed per sample using CometScore or equivalent software.

### 2.9 Statistical analysis

All quantitative data are expressed as mean ± standard deviation (mean ± SD). Data were analyzed using SPSS 26.0 software and GraphPad Prism. After confirming normal distribution, one-way analysis of variance (One-way ANOVA) was performed, followed by Tukey’s post-hoc test for multiple comparisons. Exact P-values are reported. Differences were considered statistically significant at P < 0.05. For the comet assay, 100 cells were randomly selected per sample (n = 10 rats/group) to minimize inter-animal variability.

## 3 Results

### 3.1 Testicular organ index

Compared with Group A (control), the left testicular index showed no significant change in Group B (sham-operated) (P > 0.05). The left testicular index was significantly reduced in Group C (VC model) (P < 0.001). L-carnitine treatment (Group D) significantly increased the left testicular index compared with Group C (P < 0.001), while no difference was observed between Group D and Group A (P > 0.05) (Figure 1).

**FIGURE 1:**
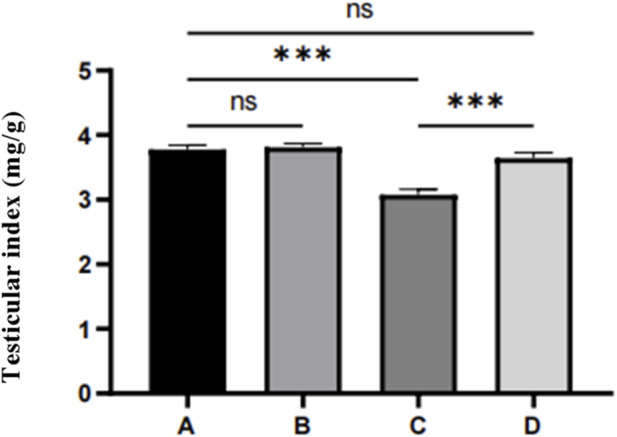
Left testicular organ index in rats from different groups. **(A)** Control group; **(B)** Sham-operated group; **(C)** Varicocele (VC) model group; **(D)** Levocarnitine-treated VC group. Data are expressed as mean ± SD (n = 10 per group). ***P < 0.001 vs. Group **(C)**.

### 3.2 Sperm quality test results

Semen parameters in Group B (sham-operated) showed no significant differences compared with Group A (control) (all P > 0.05). Compared with Groups A/B, Group C (VC model) exhibited a significant decrease in relative sperm count, progressive motility (PR), and total motility (all P < 0.001). L-carnitine treatment partially restored sperm quality: compared with Group C, Group D showed increased relative sperm count (P < 0.05), PR (P < 0.001), and total motility (P < 0.001). However, relative sperm count (P < 0.01), PR (P < 0.001), and total motility (P < 0.001) in Group D remained significantly lower than those in Group A (Figure 2).

**FIGURE 2:**
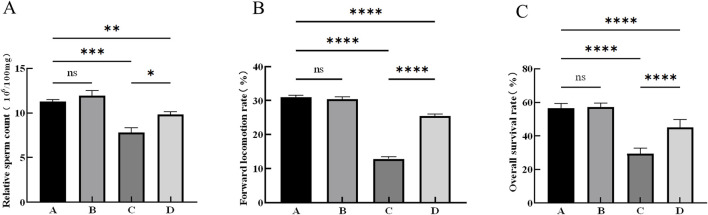
Sperm quality parameters in rats from different groups. **(A)** Relative sperm count; **(B)** Progressive motility (PR%); **(C)** Total motility. Groups: (A) Control; (B) Sham-operated; (C) Varicocele model; (D) Levocarnitine-treated VC group. Data are expressed as mean ± SD (n = 10 per group). *P < 0.05, ***P < 0.001.

### 3.3 Testicular tissue morphology under light microscope

HE staining revealed intact seminiferous tubule basement membranes in Group A (control), with complete spermatogenic cell layers including spermatogonia, spermatocytes, and mature spermatids in proper histological arrangement. The number of sperm in the lumen was large, and the supporting cells and spermatogenic cells were arranged neatly and closely, about 5-7 layers, Johnsen score (8.7 ± 0.900) points; there was no significant difference in testicular tissue morphology between group B and group A under light microscope, and there was no significant difference in Johnsen score (8.5 ± 0.806) (*P* > 0.05). The level of spermatogenic cells and the number of sperm cells in the lumen of the testis in the C group were significantly lower than those in the A group. The spermatogenic tubules were loosely arranged, the cells were disordered, and a large number of spermatogenic cells fell off. The Johnsen score (4.7 ± 0.900) was lower than that in the A group, and the difference was statistically significant (*P* < 0.001). In group D, the basement membrane of testicular seminiferous tubules was intact, the spermatogenic cells were clear and closely arranged, and mature sperm could be seen in the center of the lumen. The Johnsen score (7.1 ± 1.136) was lower than that in group A (*P* < 0.05) and higher than that in group C (*P* < 0.001) (Figures 3, 4).

**FIGURE 3:**
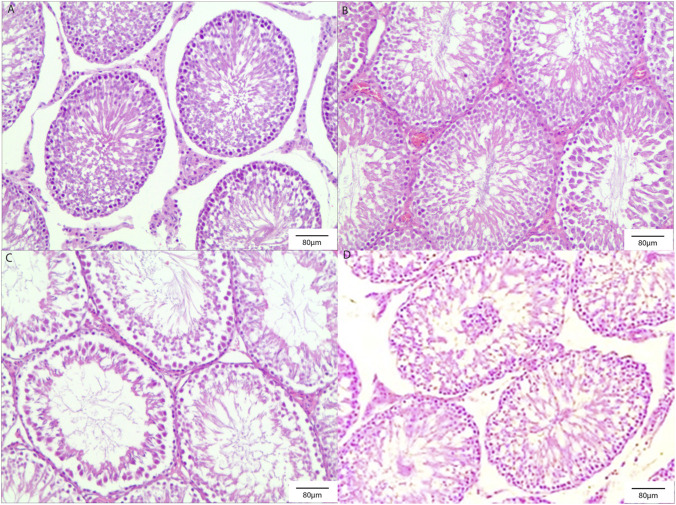
Representative photomicrographs of hematoxylin-eosin (HE) stained left testicular tissue in rats (original magnification ×200). **(A)** Control group: normal seminiferous tubule architecture with orderly arranged spermatogenic cells (5–7 layers) and abundant sperm in the lumen; **(B)** Sham-operated group; **(C)** Varicocele model group: disordered spermatogenic epithelium, germ cell sloughing, reduced sperm in lumen, and tubular atrophy; **(D)** Levocarnitine-treated group: significantly improved tubular structure and spermatogenesis. Arrowheads indicate sloughed germ cells; asterisks indicate sperm in the tubular lumen. Scale bar = 50 μm.

**FIGURE 4:**
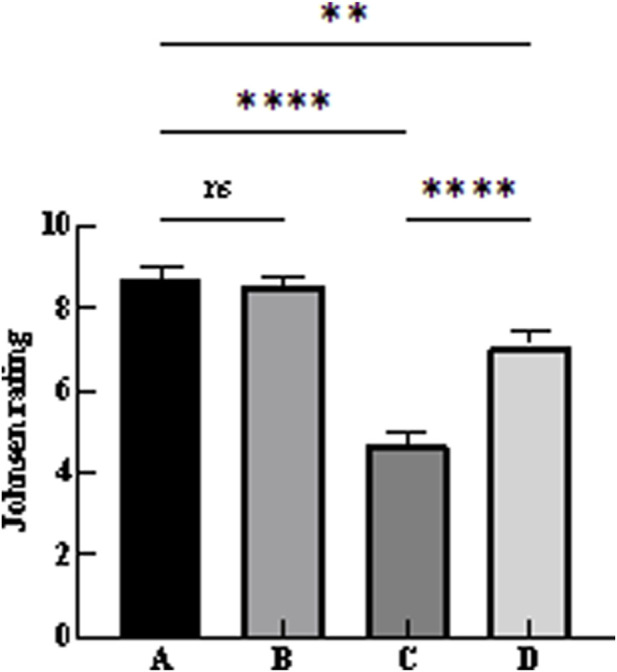
Johnsen’s score of testicular spermatogenesis in each group. Data are presented as mean ± SD (n = 10 per group). ***P < 0.001 vs. Group C; *P < 0.05 vs. Group A.

### 3.4 Test results of oxidative stress level in testicular tissue

No significant differences were observed between Group A (control) and Group B (sham-operated) in oxidative stress markers (MDA, 8-OHdG, ROS) or SOD activity in left testicular tissue (all P > 0.05). Compared with Group A, Group C showed significantly increased MDA, 8-OHdG and ROS levels (P < 0.001) and decreased SOD activity (P < 0.001). Compared with Group C, Group D exhibited significantly reduced MDA, 8-OHdG and ROS levels (all P < 0.05) and increased SOD activity (P < 0.001). However, compared with Group A, Group D still showed higher MDA and 8-OHdG levels (P < 0.05), lower SOD activity (P < 0.05), while ROS levels were not significantly different (P > 0.05) (Figure 5).

**FIGURE 5:**
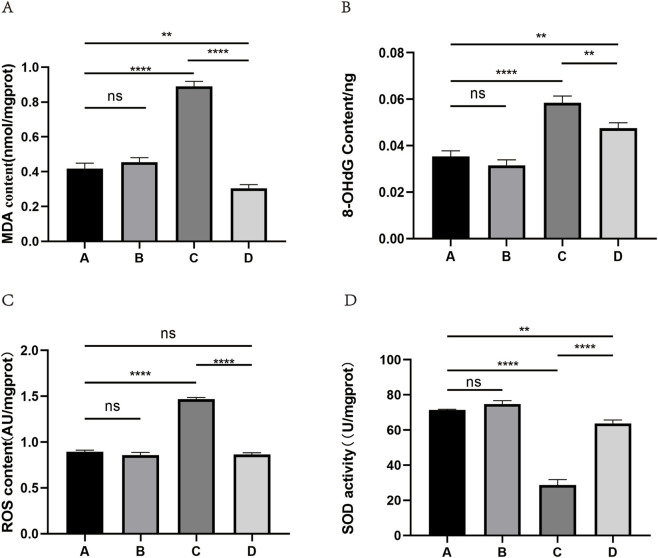
Oxidative stress markers and antioxidant enzyme activity in left testicular tissue. **(A)** MDA content; **(B)** 8-OHdG level; **(C)** ROS level; **(D)** SOD activity. Groups: (A) Control; (B) Sham; (C) Varicocele model; (D) Levocarnitine-treated group. Data are expressed as mean ± SD (n = 10 per group). *P < 0.05, ***P < 0.001.

### 3.5 FoxO3a, CAT, SOD2 protein expression in testis tissue

Compared with the control group (Group A), the expression levels of FoxO3a, CAT, and SOD2 proteins in the sham-operated group (Group B) showed no significant differences (all P > 0.05). In the varicocele model group (Group C), total FoxO3a protein expression was significantly increased (P < 0.001), whereas CAT (P < 0.01) and SOD2 (P < 0.001) protein levels were decreased compared with Group A. Notably, the level of phosphorylated FoxO3a (p-FoxO3a at Ser253) was also significantly elevated in Group C, resulting in an increased p-FoxO3a/total FoxO3a ratio (P < 0.01), suggesting suppressed transcriptional activity of FoxO3a. In the levocarnitine-treated group (Group D), total FoxO3a expression was further upregulated (P < 0.01 vs. Group C), while p-FoxO3a expression was significantly decreased (P < 0.05 vs. Group C), leading to a marked reduction in the p-FoxO3a/total FoxO3a ratio. These changes were accompanied by significantly increased expression of downstream targets CAT (P < 0.001) and SOD2 (P < 0.001) compared with Group C (Figure 6).

**FIGURE 6:**
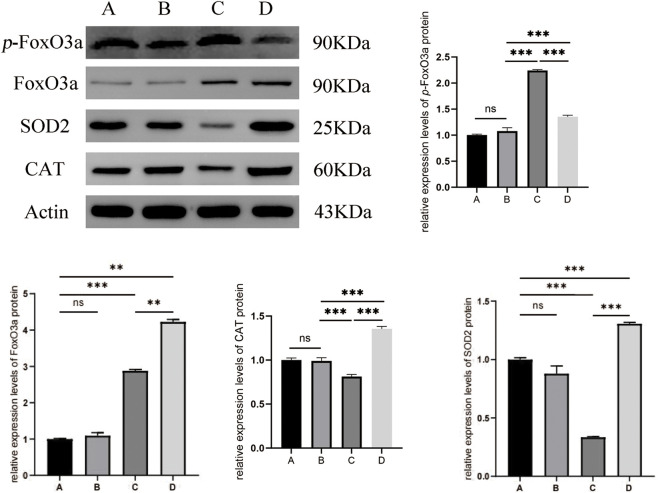
Western blot analysis of p-FoxO3a, FoxO3a, CAT, and SOD2 protein expression in rat testicular tissue. **(A)** Representative Western blot bands; **(B–D)** Densitometric quantification of FoxO3a, CAT, and SOD2, respectively. β-actin was used as the loading control. Groups: (A) Control; (B) Sham; (C) Varicocele model; (D) Levocarnitine-treated group. Data are expressed as mean ± SD (n = 6–8 per group). **P < 0.01, ***P < 0.001.

### 3.6 Morphological structure of spermatogenic cells and sertoli cells under electron microscope

Transmission electron microscopy (TEM) revealed comparable ultrastructural morphology between spermatogonia and Sertoli cells within the same experimental group. Light microscopy revealed well-defined cellular architecture in Group A, with intact plasma membranes observed in both spermatogonia and Sertoli cells of the left testis. The nucleus was slightly irregular, the nuclear membrane was intact, and the perinuclear space was not significantly widened. Mitochondria exhibited normal morphology with well-organized, evenly distributed tubular cristae. The rough endoplasmic reticulum (RER) exhibited characteristic flattened cisternae morphology without evident dilation. There was no hypertrophy and expansion of Golgi capsule; no obvious autophagy was observed in the cells. In group B, there was no obvious edema in the left testicular spermatogonia and Sertoli cells. The cell membrane was clear and complete, the cytoplasm was uniform, the nucleus was oval, the nuclear membrane was complete, and the chromatin was evenly distributed. No swelling of mitochondria, mitochondrial cristae evenly distributed; rough endoplasmic reticulum slightly expanded; no obvious autophagy was observed. In group C, the left testicular cells of rats were obviously edematous, and the cell membrane was intact and continuous. Mitochondria were severely swollen, the matrix became shallow and partially dissolved, some mitochondria were severely vacuolated or even ruptured, and a large number of mitochondrial cristae were broken and missing. Rough endoplasmic reticulum and Golgi moderate expansion. In group D, the cytoplasm of left testicular cells was slightly edematous, and the cell membrane was intact and continuous. The nuclear membrane was intact and the chromatin was evenly distributed. Mitochondria were slightly swollen, the matrix was uneven, and the mitochondrial cristae were broken and shortened. The rough endoplasmic reticulum was slightly dilated and some membranes were dissolved; the morphology of Golgi apparatus was acceptable, and no hypertrophy and hyperplasia were observed (Figure 7).

**FIGURE 7:**
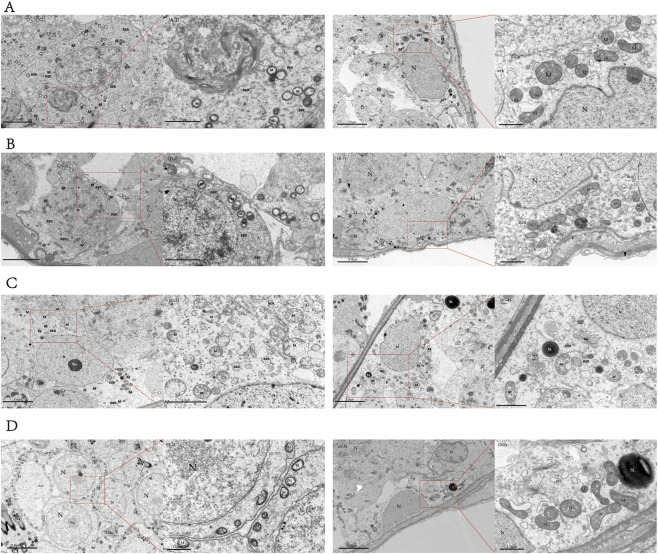
Transmission electron microscopy (TEM) images showing ultrastructural changes in spermatogenic cells and Sertoli cells of the left testis. Original magnification ×8000–12000. Scale bar = 1 μm. **(A)** Control group: normal mitochondria with intact cristae (arrow), regular rough endoplasmic reticulum (RER), and nucleus; **(B)** Sham-operated group; **(C)** Varicocele model group: severe mitochondrial swelling and vacuolization (hollow arrows), disrupted cristae, and dilated RER; **(D)** Levocarnitine-treated group: markedly improved mitochondrial morphology with less swelling and better-preserved cristae. Arrowheads indicate mitochondria; hollow arrows indicate swollen/vacuolated mitochondria; double arrows indicate RER.

### 3.7 Testicular single cell comet electrophoresis results

Tail length (TL) and olive tail moment (OTM) showed no significant differences between Group A and Group B (both P > 0.05). Varicocele induction (Group C) significantly increased TL and OTM compared with Group A (both P < 0.001). Compared with Group C, L-carnitine treatment (Group D) significantly decreased OTM (P < 0.05), whereas TL remained unchanged (P > 0.05) (Figures 8, 9).

**FIGURE 8:**
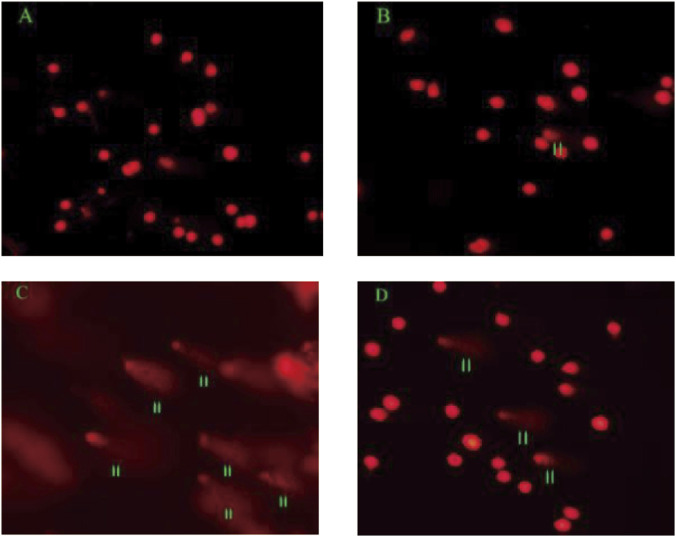
Representative fluorescence images of single-cell comet assay in testicular cells from each group. **(A)** Control group; **(B)** Sham-operated group; **(C)** Varicocele model group; **(D)** Levocarnitine-treated group. Increased comet tails indicate DNA damage.

**FIGURE 9:**
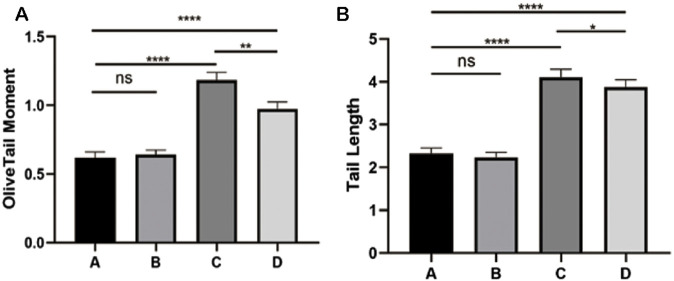
Quantitative analysis of DNA damage in testicular cells by comet assay. **(A)** Tail length (TL); **(B)** Olive tail moment (OTM). Groups: (A) Control; (B) Sham; (C) Varicocele model; (D) Levocarnitine-treated group. Data are expressed as mean ± SD (100 cells per sample, n = 10 rats per group). *P < 0.05, ***P < 0.001.

## 4 Discussion

Male infertility caused by varicocele accounts for about 40% of the infertile population, and has become the most common corrective cause of male infertility (Sasson and Kashanian, 2020). Studies have shown that oxidative stress is the main cause of male infertility (Garolla et al., 2015; Ammar et al., 2021; Signorini et al., 2024). L-carnitine is an endogenous non-essential branched-chain amino acid derivative with antioxidant effect. It can be synthesized by multiple organs such as liver, kidney, and testis. It participates in lipid metabolism by transporting fatty acids from the cytoplasm to mitochondria for β-oxidation. Studies have shown that L-carnitine can also be used as a cell protective agent to remove excessive reactive oxygen species produced in the body, play an anti-inflammatory and antioxidant role, thereby maintaining cell membrane stability, maintaining cell DNA integrity, and promoting DNA repair (Chang et al., 2021).

The organ index refers to the ratio of animal organ weight to body weight, which can reflect the development and damage of organs to a certain extent (Mogheiseh et al., 2022; Bercovitch and Rodriguez, 1993; Erthal et al., 2022; Li et al., 2018; Hwang et al., 2011). In this study, the organ index of testis and epididymis and the sperm motility of rats in each group were measured. It was found that varicocele caused a decrease in the weight of testis and epididymis, resulting in a decrease in the number and motility of sperm, while L-carnitine significantly improved these impairments, suggesting a partial restoration of reproductive parameters.

After lipid and DNA oxidative damage in cells, peroxidation products such as MDA and 8-OHdG will be produced. The content of MDA and 8-OHdG can be detected to determine the degree of damage of oxygen free radicals to cell membrane and chromatin *in vivo* and to monitor the damage of oxidative stress to spermatogenic cells (Fan et al., 2020; Manisaligil et al., 2022; Wei et al., 2019). ROS can directly reflect the level of oxidative stress *in vivo*. Excessive ROS will have toxic effects on sperm, and SOD is one of the main antioxidant enzymes in cells. It is an important protective factor to inhibit the damage of peroxidation to spermatogenic cells *in vivo* (Zhang et al., 2022; Yousefi et al., 2022; Sun et al., 2022; Ammar et al., 2022). In this study, it was found that artificial varicocele in male Wistar rats could increase the levels of MDA and 8-OHdG and ROS activity in the testis of rats, while the activity of SOD was significantly decreased. This indicates that varicocele may increase the level of oxidative stress in the testis, increase the damage of peroxidation to spermatogenic cells, and also consume the antioxidant enzyme SOD to reduce its activity. After treatment with exogenous L-carnitine, the levels of MDA and 8-OHdG and ROS activity in the testis of rats with varicocele were significantly decreased, and the activity of SOD was significantly increased, which indicated that L-carnitine had a certain protective effect on testicular peroxidation caused by varicocele.

Oxidative stress, DNA damage and other factors activate FoxO through a variety of upstream signaling pathways, and then activate FoxO protein, exerting anti-oxidation, regulation of cell cycle and autophagy by regulating downstream genes (Eijkelenboom and Burgering, 2013; Martins et al., 2016). FoxO3a is a member of the Forkhead box O (FoxO) transcription factor family. Its function is regulated by post-translational modifications and it can control the expression of mitochondrial antioxidant enzymes such as SOD2 and CAT, thereby contributing to cellular redox homeostasis. In this study, the protein levels of FoxO3a, CAT and SOD2 in the testis of rats in each group were detected by Western blot. It was found that varicocele could increase the expression of FoxO3a in the testis of rats, while the expression of CAT and SOD2 decreased. When the reactive oxygen species were continuously excessive, CAT and SOD2 were not enough to completely remove the excessive ROS, the spermatogenic cells and Sertoli cells of the testis were oxidatively damaged. Exogenous supplementation of L-carnitine could mediate the enhancement of FoxO signaling pathway and increase the expression level of FoxO3a. In turn, more CAT and SOD2 are produced to remove excessive ROS in mitochondria, thereby improving the antioxidant capacity of testicular tissue and supporting the preservation of reproductive function. The activity of FoxO3a is primarily negatively regulated by Akt-mediated phosphorylation at Ser253, which promotes its nuclear exclusion and cytoplasmic degradation. In the present study, levocarnitine treatment reduced p-FoxO3a (Ser253) levels while increasing total FoxO3a expression, thereby enhancing the nuclear translocation capacity of FoxO3a. This finding is consistent with previous reports showing that antioxidants can activate the FoxO3a pathway by inhibiting the PI3K/Akt signaling cascade, ultimately promoting the transcription of antioxidant enzymes such as CAT and SOD2 (Hagenbuchner and Ausserlechner, 2013).

Mitochondria provide energy for sperm cells through oxidative phosphorylation, and also produce endogenous ROS. Mitochondrial DNA is more susceptible to oxidative damage due to its proximity to the oxidative phosphorylation site of the mitochondrial inner membrane, and long-term use of mitochondrial targeted antioxidants can reduce mitochondrial DNA damage. (Williamson et al., 2020). In this study, it was found that the number of mitochondria in germ cells of rats in the varicocele model group decreased, and the mitochondrial cristae became shorter or even disappeared, which would greatly reduce the ability of germ cells to synthesize ATP, cause energy supply disorder, and eventually lead to oligoasthenospermia. The use of sufficient L-carnitine treatment found that the oxidative damage of organelle structure in spermatogenic cells was significantly improved, indicating that L-carnitine can effectively maintain the structural integrity of organelles, especially mitochondria, in testicular tissue.

On the other hand, this study detected the degree of DNA damage in testicular cells by single cell comet electrophoresis and found that compared with the control group, TL and OTM in spermatogenic cells of rats in the model group increased significantly, and OTM in spermatogenic cells of rats in the L-carnitine group was significantly lower than that in the model group, but the difference in TL was not statistically significant. This indicates that varicocele can cause severe DNA damage in testicular spermatogenic cells, and exogenous addition of L-carnitine has a certain protective effect on oxidative stress caused by varicocele, which can protect testicular cell DNA from oxidative damage. Once DNA damage has occurred, the effect of L-carnitine on protecting DNA from further damage may be limited.

Although our results demonstrate that levocarnitine treatment significantly upregulated the expression of FoxO3a, CAT, and SOD2, and improved oxidative stress markers and mitochondrial ultrastructure, the present study only assessed total FoxO3a protein levels. We did not examine the phosphorylation status of FoxO3a or perform loss-of-function experiments (e.g., FoxO3a inhibitors or siRNA). Therefore, the data support an association rather than a definitive causal role of the FoxO3a pathway in the protective effects of levocarnitine. Further mechanistic studies are warranted to confirm this pathway’s direct involvement. This study employed a surgically-induced varicocele model in male Wistar rats to investigate testicular dysfunction and therapeutic interventions. It was found that the varicocele model could reduce the testicular index of male Wistar rats, reduce the total sperm viability, forward movement rate and relative sperm count, increase the level of oxidative stress in the testis, weaken the antioxidant capacity, and cause testicular tissue morphological damage and structural damage of organelles in Sertoli cells and spermatogenic cells. L-carnitine treatment may increase the expression of CAT and SOD2 in testicular tissue by mediating the activation of FoxO signaling pathway, improve the antioxidant capacity of rat testis, reduce the oxidative damage of testis in Wistar male rats with varicocele, and thereby improve testicular function and partially restore reproductive function in rats with varicocele.

## 5 Limitations

Several limitations should be acknowledged in this study. First, levocarnitine was administered via intraperitoneal injection at 100 mg/kg/day, whereas clinical use in humans is typically oral with much lower relative doses (usually 1–3 g/day). Due to differences in metabolism and bioavailability between rats and humans, the translational potential of the current dosage requires caution. Second, although transmission electron microscopy showed improvement in mitochondrial ultrastructure, direct assessments of mitochondrial function—including mitochondrial membrane potential, mitochondrial-specific ROS production, and ATP levels—were not performed. These measurements would provide stronger evidence for mitochondrial protection. Finally, the relatively short 28-day observation period limits conclusions regarding long-term efficacy and safety. Future studies should incorporate clinically relevant dosing regimens and more comprehensive mitochondrial functional assays.

## Data Availability

The original contributions presented in the study are included in the article/supplementary material, further inquiries can be directed to the corresponding author.
